# A computational passage-of-time model of the cerebellar Purkinje cell in eyeblink conditioning

**DOI:** 10.3389/fncom.2023.1108346

**Published:** 2023-03-06

**Authors:** Matthew Ricci, Junkyung Kim, Fredrik Johansson

**Affiliations:** ^1^Carney Institute for Brain Sciences, Brown University, Providence, RI, United States; ^2^Department of Experimental Medical Science, Lund University, Lund, Sweden

**Keywords:** Purkinje cell, timing, associative learning, eyeblink conditioning, cerebellum

## Abstract

The cerebellar Purkinje cell controlling eyeblinks can learn, remember, and reproduce the interstimulus interval in a classical conditioning paradigm. Given temporally separated inputs, the cerebellar Purkinje cell learns to pause its tonic inhibition of a motor pathway with high temporal precision so that an overt blink occurs at the right time. Most models place the passage-of-time representation in upstream network effects. Yet, bypassing the upstream network and directly stimulating the Purkinje cell's pre-synaptic fibers during conditioning still causes acquisition of a well-timed response. Additionally, while network models are sensitive to variance in the temporal structure of probe stimulation, *in vivo* findings suggest that the acquired Purkinje cell response is not. Such findings motivate alternative approaches to modeling neural function. Here, we present a proof-of-principle model of the passage-of-time which is internal to the Purkinje cell and is invariant to probe structure. The model is consistent with puzzling findings, accurately recapitulates Purkinje cell firing during classical conditioning and makes testable electrophysiological predictions.^1^

## 1. Introduction

While long-term potentiation (LTP) and long-term depression (LTD) are often measurable during memory encoding, the prevailing dominant view that such changes in synaptic strength entirely explain memory has been challenged by several findings. In both Aplysia (Chen et al., 2014) and mammalian hippocampal cells (Ryan et al., 2015), long-term memory does not in all cases require the persistence of changes in synaptic strength. Furthermore, results from eyeblink conditioning suggests that a single Purkinje cell can memorize a temporal relationship of hundreds of milliseconds between two inputs (Johansson et al., 2014).

**Figure 1:**
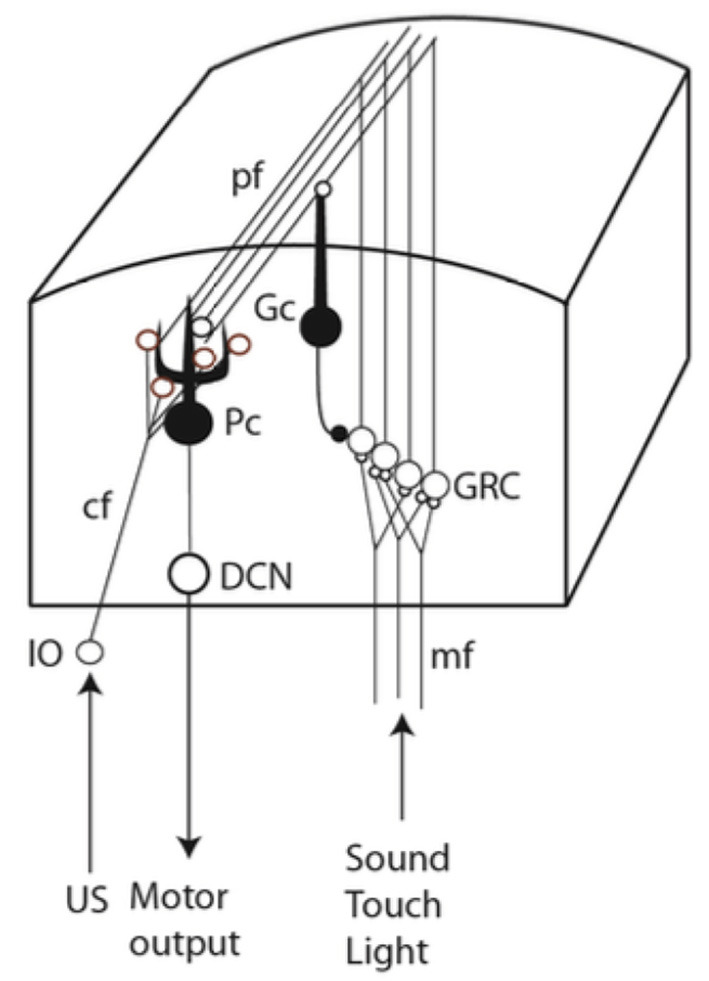
Simplified neural circuitry. The CS is transmitted *via* the mossy/parallel fiber system and the US *via* the climbing fiber. IO, inferior olive; cf, climbing fiber; mf, mossy fibers; Pc, Purkinje cell; Gc, Golgi cell; pf, parallel fibers; GRC, granule cells; DCN, deep cerebellar nuclei.

If a conditional stimulus (CS), such as a tone, is repeatedly followed by a blink-eliciting unconditional stimulus (US), with a fixed temporal delay (the interstimulus interval or ISI), a blink response to the CS develops. This conditioned response (CR) occurs just before the expected US onset for ISIs from 100 ms to seconds (Gormezano and Moore, 1969; Kehoe and Macrae, 2002). The underlying learning occurs in a specific physiologically defined microzone of cerebellar cortex which demonstrably controls the eyelid (Yeo et al., 1985a; Hesslow, 1994a,b; Mostofi et al., 2010; Heiney et al., 2014). Because many and diverse conditional stimuli may predict the same unconditional stimulus, the association-forming mechanism should exhibit extensive fan-in, which the Purkinje cell indeed does; its vast dendritic arbor gets CS input from ~200,000 parallel fibers (Harvey and Napper, 1991) that come from the tiny and densely packed granule cells (GRC) in the granular layer of the cerebellar cortex, while it gets US input from a single climbing fiber (Yeo et al., 1985b; Mauk et al., 1986; Steinmetz et al., 1989; Hesslow and Yeo, 2002; Figure 1) that contacts the entire dendritic arbor (Eccles et al., 1967).

Purkinje cells, which are the sole output neurons of the cerebellar cortex, have high tonic firing rates due to an internal pacemaker mechanism (Cerminara and Rawson, 2004). Given CS-US pairings, blink-controlling Purkinje cells learn an adaptively timed pause response (Hesslow and Ivarsson, 1994; Jirenhed et al., 2007; Jirenhed and Hesslow, 2011a; Halverson et al., 2015; Ten Brinke et al., 2015) in their tonic inhibition of a motor pathway (Hesslow and Yeo, 2002). This learned pause mirrors the known features of behavioral CRs. It is extinguished during repeated CS alone presentations and it is rapidly re-acquired (Jirenhed et al., 2007). The pause is always adaptively timed in that it reaches its maximal amplitude just before the predicted onset of the US, and ends shortly after, even if the CS lasts only a few milliseconds or outlasts the ISI by hundreds of milliseconds (Jirenhed and Hesslow, 2011b; Johansson et al., 2014). In temporal uncertainty paradigms where mixed trials of different ISIs are used, multiple temporally locked pause responses to the same CS are learned (Halverson et al., 2015; Jirenhed et al., 2017). Importantly, learning at the behavioral level is unreliable or absent with short ISIs (Smith et al., 1969; Salafia et al., 1980). A similar minimal ISI of 100 ms for effective conditioning is observed at the level of the single Purkinje cell as well (Wetmore et al., 2014).

Contemporary modeling is dominated by the idea of the granular layer network generating a passage-of- time (POT) representation. (Bullock et al., 1994; Buonomano and Mauk, 1994; Medina and Mauk, 2000; Hansel et al., 2001; Yamazaki and Tanaka, 2009; Lepora et al., 2010). Before learning, the Purkinje cell is assumed to fire at its tonic rate due to net zero or net excitation from the balanced activity of excitatory granule cells and inhibitory basket/stellate interneurons. In response to a CS drive from the mossy fibers, time-variance in granule cell (GRC) activity is in most models assumed to arise from fast and partly lateral feedback inhibition from Golgi cell interneurons (reviewed in Johansson et al., 2016). The effect would be a series of random transitions between activity and quiescence in each granule cell, creating instantaneous population vectors that represent time. LTD is then selectively recruited for those GRC-to-Purkinje cell synapses most activated by the CS around the time of US onset. LTD tips the balance of afferent activity toward net inhibition near expected US onset, so that, after learning, the CS alone initiates a well-timed pause in Purkinje cell firing.

It is challenging to reconcile granule cell POT models with several empirical results. First, Purkinje cells learned well-timed pause responses to different ISIs even though the granular layer network was bypassed (Johansson et al., 2014). Furthermore, both GABA-ergic interneurons (Johansson et al., 2014) and glutamatergic AMPA receptors in the molecular layer (Johansson et al., 2015) could be blocked without disrupting the pause response. These three puzzling findings are independently difficult to reconcile with current modeling. Instead, these results suggest that the precisely timed Purkinje cell activity could depend upon an internal timing mechanism that measures and stores temporal duration.

Furthermore, existing POT models require the probe stimulus to endure for at least as long as the training stimulus. For, as soon as the probe terminates, afferent activity would shift to its pre-stimulation conditions and return the net input of the Purkinje cell to net zero, preventing pause expression. However, after training, the Purkinje cell and experimental subject can produce a well-timed pause and overt blink given only the initial (< 20 ms) part of the CS (Svensson and Ivarsson, 1999; Jirenhed and Hesslow, 2011b). Also, Johansson et al. (2014) found that drastically varying the CS on probe trials (17.5–800 ms, 100–400 Hz) had no effect on the learned pause. This suggests that the computational process underlying the CR contains a strong non-linearity which switches the Purkinje cell from a tonic spiking state to a pause-expressing state.

Biochemical models that present possible mechanisms for parts of the phenomena have recently been proposed (Majoral et al., 2020; Mandwal et al., 2021). Given that the currents and molecules involved are yet to be elucidated, here we explore a conceptual and mathematical model of what kind of mechanism could explain all the phenomena described above.

We see two crucial criteria for the modeling of a Purkinje cell timing mechanism:

- Physiological: The neural machinery for learning and expression of conditioned pause responses must be contained in the Purkinje cell itself.
- Computational: The computational process initiating expression of a pause response must be gated by a strong non-linearity or “switch.”

The model is inspired by a suggested conceptual mechanism whereby CS onset prompts the Purkinje cell to release a batch of evolving “recorder units” (Johansson and Hesslow, 2014). The precise biophysical identity of recorder units does not substantially affect the abstract theory; the only crucial feature of these units is that they evolve through different time-encoding states beginning at CS onset. Each unit contributes to inhibition of the cell at the interval that it encodes, and the strength of inhibition at any given time after CS onset is encoded by the quantity of units that encode the same interval, which increases *via* repeated exposure to CS-US pairs. Recorder units thus constitute an intracellular POT representation. Importantly, this idea satisfies the two above criteria: the pause mechanism is internal to the Purkinje cell and it is mediated by a switch.

Below, this theory is instantiated as a computational model with three parts. A “write” module intracellularly records the interstimulus interval. The distribution of recorded intervals is stored in an “archive.” Third, a “read” module translates the archived intervals into a timed hyperpolarization event. These states become active only when afferent activity switches the cell from its passive OFF state to its active ON state. We trained this model cell with several paradigms typical to the eyeblink conditioning literature and found:

- Given ISIs ranging from 100 to 500 ms, the cell learns a pause response with critically timed onset, maximum and offset, as in Jirenhed et al. (2007) and Johansson et al. (2014).
- After training, the cell produces the learned pause given only the initial part of the CS, as in Svensson and Ivarsson (1999), Jirenhed and Hesslow (2011b), and Johansson et al. (2014).
- The learned pause is extinguished by CS-only trials in about as much time as initial acquisition, as in Jirenhed et al. (2007).
- Alternating ISIs across trials teaches the cell two pauses, as in Jirenhed et al. (2017).

Additionally, we ran simulations, which, to our knowledge, have not been tested on the biological Purkinje cell. First, we tried stimulating the cell with two-part CSs or two-part USs so that the cell was effectively exposed to two ISIs on each trial. If the CS consisted of two impulse trains separated by a period of silence followed by a US, the model cell learned the longer ISI. If two USs were presented, the model cell learned the shorter ISI. This contrasts with established models. Next, we tried varying the intertrial interval (ITI) along with the ISI. We found that time to pause acquisition in all simulations was a function of both of these temporal quantities.

The computational model reproduces the surprising findings in Johansson et al. (2014) and accords well with the conditioning literature at large. Additionally, the model displays several behaviors thus far not explained by existing models. We concentrate on the formal aspects of the model and their empirical motivation, rather than on biophysics. While many details of the molecular basis for this phenomenon remain unknown and thus preclude the latter kind of model, we believe that our computational simulation and predictions can be informative. Most striking among the results is a dependency of the number of trials until pause acquisition on the ISI/ITI ratio. The behavior of this formal model may help to inform future electrophysiology.

## 2. Model

### 2.1. Model structure

**Figure 2:**
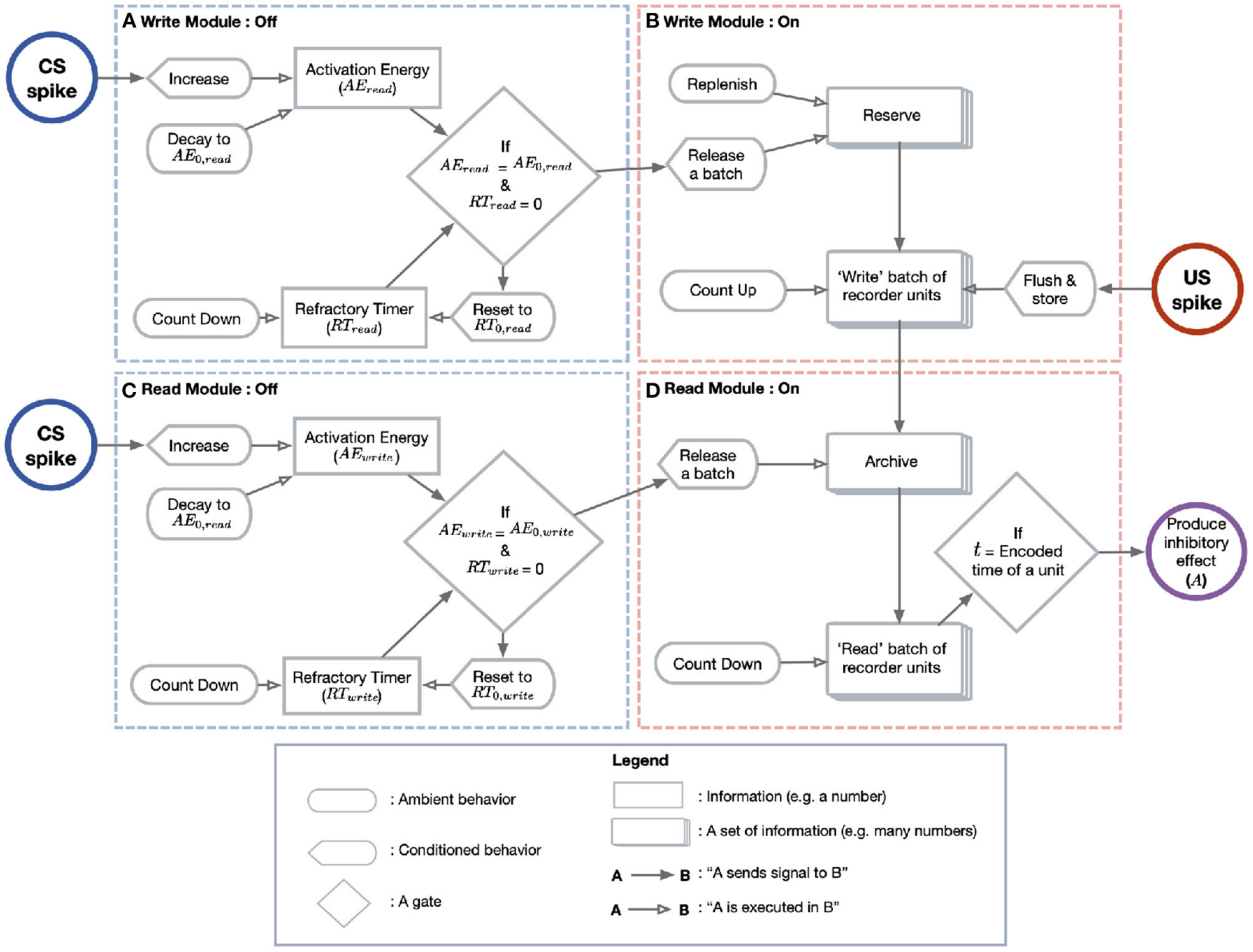
Model diagram. This diagram depicts the flow of events leading to the CR in the model Purkinje cell. **(A)** The write module's switch is controlled by the quantity AEwrite (upper box). AEwrite is increased with each CS spike (blue circle) and is otherwise subject to a passive decay. Only when AEwrite reaches a threshold (diamond) does the cell transition to the write/ON state. When the switch is activated, a refractory timer (lower box) is reset and begins to tick downward until it reaches 0. **(B)** When the write module switches ON, the reserve (tiled boxes, top) continually ejects a batch of recorder units (tiled boxes, bottom). The reserve is then replenished until at maximum capacity. The ejected recorder units advance in state with the passage of time until the first US spike (red circle), at which point the batch is stored in the archive. **(C)** The switch on the read module behaves exactly as in the write case, but with different parameter values. **(D)** A constant fraction of the archive (tiled boxes, top) is sampled. Each unit “counts down” until the time encoded by its state. At this time (diamond), the unit introduces a fixed inhibitory current to the membrane (purple circle).

The Purkinje cell has two computational goals. First, it must record or “write” an ISI into memory, here instantiated as an “archive.” Second, it must “read” this ISI into a well-timed pause response. As stated above, the Purkinje cell only transitions from its passive spiking behavior to its active read/write behavior when some type of switch is activated. Thus, we may formally model the cell as having two modules (read/write; Figure 2, top vs. bottom row) which can be in two states (OFF/ON; Figure 2, left vs. right column).

Before CS stimulation, both the read and write modules are OFF and the cell spikes at its tonic rate. When pre-synaptic spikes arrive at the cell, a quantity called “activation energy” (AEwrite and AEread) begins to increase. When AEwrite reaches a threshold value, the write module switches to the ON state. A refractory timer on this switch is then reset so that the cell is insensitive to the CS for a given period.

When the write module is activated after sufficient CS stimulation, a batch of “recorder units” is released from a “reserve.” These units are a formal abstraction meant to stand for whatever process encodes the POT within the Purkinje cell. The reserve is a way of imagining these units before the write module is activated. After some recorder units have been released from the archive, the reserve begins to replenish at a constant rate. The released recorder units evolve through states in a way that mirrors the passage of time. For example, if they can encode four states, *A* = 50 ms, *B* = 100 ms, *C* = 200 ms, and *D* = 300 ms, then the POT is represented by the sequential evolution of the recorder units *A*→*B*→*C*→*D*. Units in the reserve encode 0 ms. In the model presented here the evolving steps are however continuous rather than discrete.

At US onset, the evolution stops and the batch of recorder units is stored in an “archive.” Again, this archive is simply a way of imagining the units after their evolution has been terminated by US onset (unless US onset is too early, for reasons discussed below). We assume that the evolution of the batch is noisy, so that, when the evolution stops, there are some units in each state. Thus, the stored batch is a histogram which constitutes an estimate on the ISI. For example, if the ISI is 200 ms, then there will be some units in states A, B, and C. We choose to imagine the recording medium as consisting of many individual “units,” since we want to use a stored histogram (which displays the frequencies of many elements or samples) to estimate the ISI. The routine of write/OFF → write/ON → store batch is repeated with each CS presentation, as long as they are spaced far enough apart to respect the refractory timer on the write module. With each presentation, a batch of recorder units is released, evolves and is stored in the archive, updating the total number of recorder units in the archive encoding each time value.

Parallel to the writing process is a reading process, which is itself gated by a switch mechanism (Figure 2, bottom left). Whether the read switch is activated depends on the read module's own activation energy (AEread) and refractory timer. When AEread reaches a threshold, the read module transitions to the ON state (Figure 2, bottom right). The activated reading module samples a batch of stored recorder units from the archive. Each recorder unit in this batch contributes a fixed amount of inhibition to the cell at a time corresponding to its state. For instance, if batch is released from the archive at time *t* = 0, then a unit in state A will introduce inhibition to the membrane potential at *t* = 50 ms. Note that, the more units there are in a batch encoding a particular time, the greater the inhibition will be at this time. In this way, the shape of the archive's histogram is directly translated into the timecourse of inhibition.

### 2.2. Model details

For simplicity, we model the Purkinje cell as a single-compartment, leaky integrate-and-fire-neuron (Equation 1) whose parameters are given in Table 1. This simple neural spiking model is appropriate for our purposes, since the Purkinje cell CR is postulated to be internally generated and therefore does not rely on complicated integration of pre-synaptic spikes:


(1) $$\begin{array}{l} \tau_{m}\frac{dV}{dt}=V\left(t\right)-V_{\text{rest}}+R_{e}CS\left(t\right)-R_{i}A\left(t\right)+R_{p}P\left(t\right). \end{array}$$

The membrane potential is controlled by three sources of current: *CS*, the 0–1 valued impulse train transmitted by the parallel fibers with resistance *R*_*e*_; *A*, internally-generated inhibitory current by the archive with resistance *R*_*e*_; and, *P*, internal, excitatory current generated by the cell's intrinsic pacemaker, which we model as a Poisson impulse train with resistance, *R*_*p*_, and rate, λ. When the membrane potential reaches a threshold, *V*_threshold_, the potential spikes to *V*_spike_ and then resets to *V*_hyperpolarization_.

**Table 1:** Parameters and their values for the formal passage-of-time model.

Parameter	τ_*m*_	*V*_rest_	*V*_threshold_	*V*_hyperpolarization_	*V*_spike_	*R*_*e*_	*R*_*i*_	*R*_*p*_	τ_write_	τ_read_
**Value**	5 ms	−70 mV	−54 mV	−85 mV	10 mV	50 Ω	2.25 × 10^6^ Ω	50 Ω	70 ms	200 ms
**Parameter**	*AE*_0, write_	*AE*_read_	*AE*_thresh, write_	*AE*_read, write_	*R*_max_	τ_reserve_	*q*	σ	λ	*c*
**Value**	0	0	2	2	1	100	1.25 × 10^−7^ recorder units/ms	40 ms	.3	3 × 10^−2^

The membrane potential parameters are set to standard values and to match the empirically observed tonic firing rate of the Purkinje cell.

The switches on both the write and read modules are controlled by a simple exponential growth function controlling activation energy:


(2) $$\begin{array}{l} \frac{dAE_{\text{module}}}{dt}=\frac{1}{\tau_{\text{module}}}\left(AE_{\text{module}}-AE_{0,\text{module}}\right)+CS\left(t\right) \end{array}$$

Here, *AE*_module_ refers to the activation energy on the switch for either the write or read module. The time constant for each module's switch, τ_module_, controls the rate at which the energy grows from its initial value, *AE*_0, module_, to a threshold, *AE*_thresh, module_. The binary impulse train *CS*(*t*) increments *AE*_module_ by 1 with each parallel fiber spike.

If the pre-synaptic spikes arriving at the Purkinje cell from CS stimulation are sufficiently frequent, *AE*_write_ is driven to threshold, at which point recorder units are released and allowed to evolve. The write module's reserve can hold a maximum of *R*_max_ = 1 recorder units, representing 100% fullness. When the write module switches on, the reserve depletes exponentially with time constant τ_reserve_ and its refractory timer is reset so that it cannot detect the CS for a fixed interval. As soon as the reserve begins to empty, it begins to slowly repopulate at a constant rate *q*. For simplicity, we assume that the state *s* of a released recorder unit *r* is real-valued, so that a recorder unit can encode any time between CS onset and US onset up to some maximum granularity Δ*t*.

**Figure 3:**
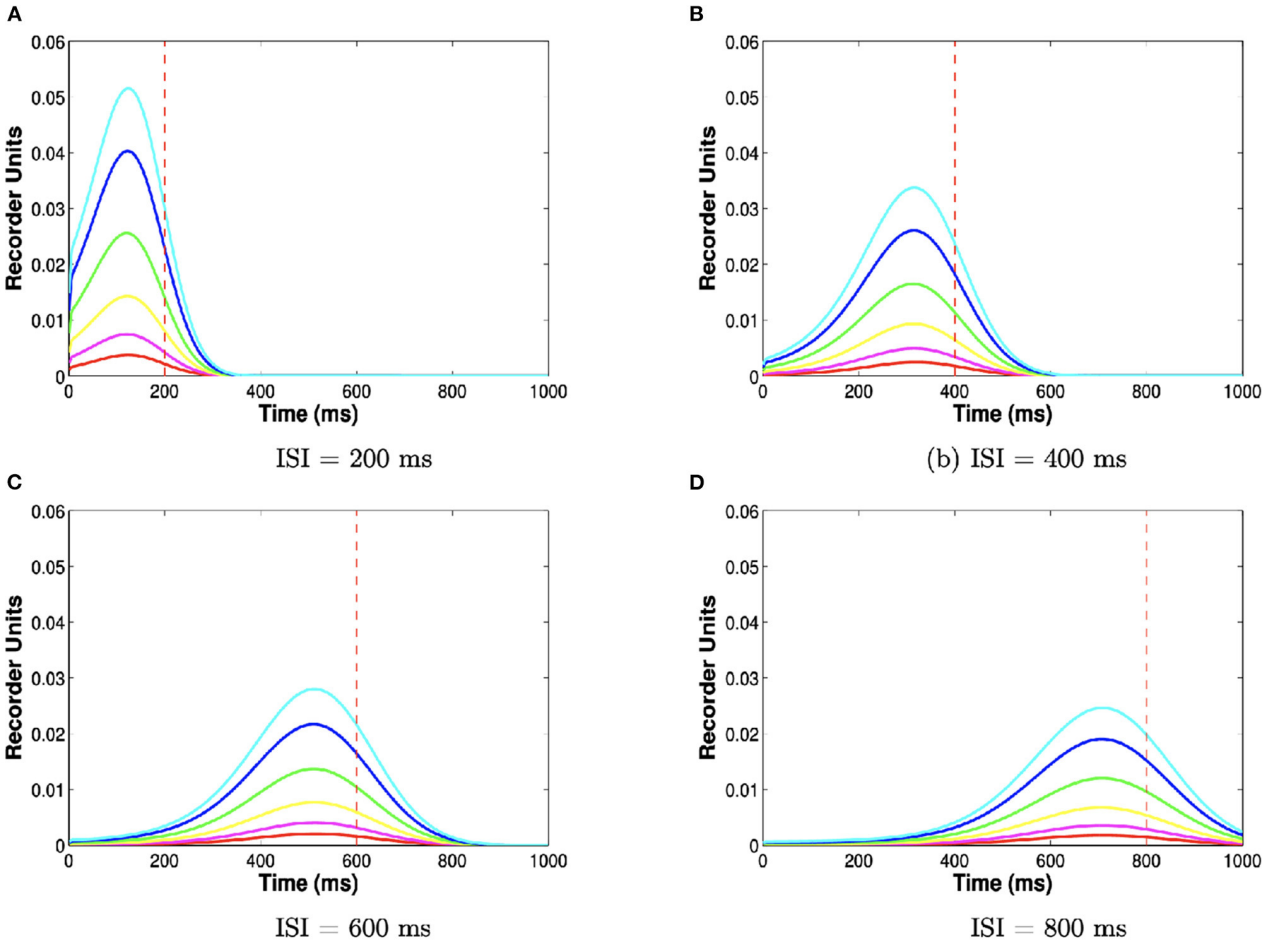
ISI and number of trials determine archive shape. Each curve plots the archive (y-axis, number of units encoding each time step on the x-axis) for different training durations and ISIs. Colors indicate 20 (red), 40 (magenta), 80 (yellow), 160 (green), 320 (blue), 640 (cyan) training trials. Further training trials make the archive grow taller. Panels indicate 200 **(A)**, 400 **(B)**, 600 **(C)**, and 800 **(D)** ms ISIs. Longer ISIs make the archive less peaked. These different shapes translate into different inhibition profiles *via* the reading module.

At the first US spike, the released batch of recorder units is added to a cumulative archive, unless US onset is < 100 ms. If the first US spike occurs before this time, we assume that the batch is simply discarded since neither the Purkinje cell pause nor the overt blink can be learned for ISIs < 100 ms. The shape of the batch, and therefore the shape of the archive, is controlled by the number of trials and the ISI (Figure 3). For short ISIs >100 ms, the stored batch will consist mostly of recorder units approximately encoding the ISI, since noise had little time to take effect. For longer ISIs, noise overwhelms the evolution of recorder units, effectively smoothing the histogram and reducing the peak near the ISI.

During the conditioning session, each CS triggers the release of evolving recorder units, to the extent that those units are available and have been replenished after previous releases, and the US freezes their evolution to be stored in a single, persistent archive. This archive, formally, is a single histogram whose mode is an estimate of the CS-US interval. Brownian noise is simulated by repeated blurring of the archive with a Gaussian kernel of standard deviation σ = 40 ms.

The read module acts in parallel to the write module. When pre-synaptic spikes are sufficiently rapid to drive AEread to threshold, the read module switches to the ON state, and a constant fraction c of recorder units are sampled from the archive. After activation, the read module's refractory timer resets so that reading is deactivated for a fixed interval. Each unit in the read batch then introduces a fixed amount of inhibitory current to the membrane at the time corresponding to its state: if read switches ON at time *t* = 0 and the archive contains *n*_*r*_ recorder units encoding state *s* = *t*_1_, then *A*(*t*_1_) = *n*_*r*_*cg*_*i*_. The shape of the stored archive (i.e., the histogram estimate on the ISI) is thereby directly translated into inhibitory current.

## 3. Results

### 3.1. Simulation 1: The cell learns a well-timed pause response

**Figure 4:**
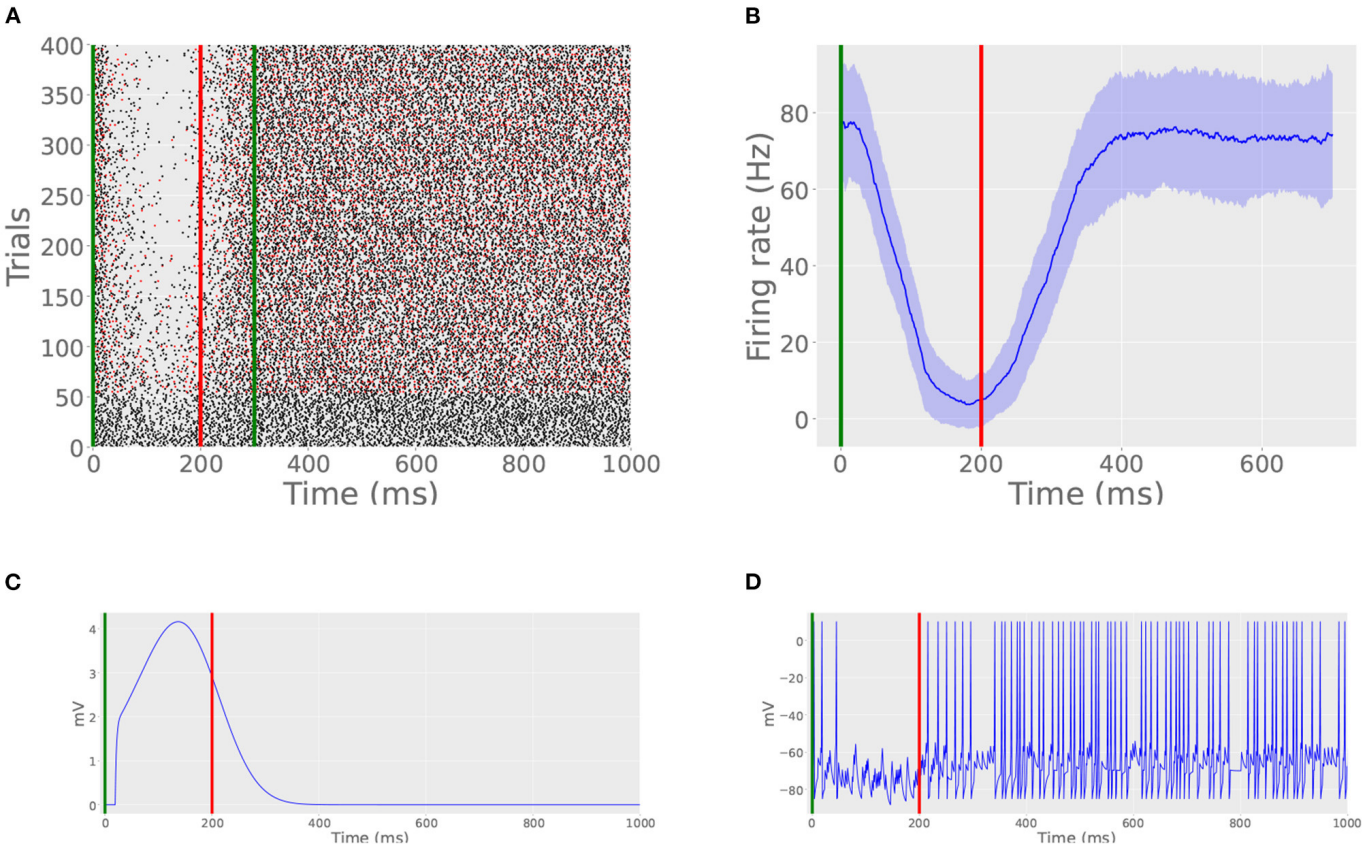
Basic CR acquisition. **(A)** Raster plot of spike times from Simulation 1. Green lines indicate CS onset and offset. Red line indicates US onset. Probe trials are marked by red spikes. Probe trials still produce the learned pause. **(B)** Peristimulus time histogram from Simulation 1. Vertical lines indicate stimulation times, as in **(A)**. **(C)** Inhibition caused by the archive on trial 400. Note that the time course of inhibition mimics the shape of the archives displayed in Figure 3A, offset by a few tens of milliseconds. This offset is caused by the time it takes for the read module to detect CS onset and initiate hyperpolarization. **(D)** Actual membrane potential during interstimulus period on trial 400. Note the thinning of spikes during the ISI, indicating a well-timed CR.

First, we trained the model cell with a 200 ms ISI (Figure 4A). CS stimulation consisted of a 100 Hz impulse train enduring for 300 ms and US stimulation consisted of a 500 Hz impulse train enduring for 20 ms and beginning at 200 ms. The cell was trained over the course of 400 trials with an ITI of 15 s. We declared that trial on which the average spiking rate during the ISI dropped to below 25% of the pre-training value to be the first CR. In this simulation, the cell learned the pause response at trial 124. Additionally, starting after 300 trials, we removed US stimulation on every 20th trial. On these probe trials (Figure 4A, red spikes), the cell still produced a pause visually indistinguishable from that of non-probe trials. An average firing rate, estimated by binning spikes in windows of 20 ms, averaging across 100 trials, is shown in Figure 4B. The inhibition created by the archive and its effect on the cell's membrane potential are shown in Figures 4C, D, respectively.

**Figure 5:**
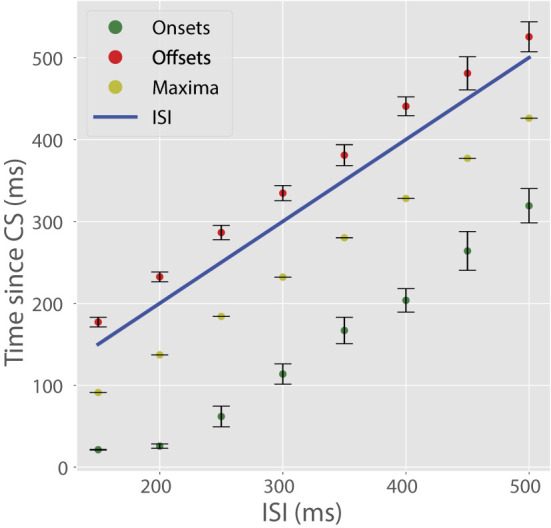
CR temporal features with varying ISIs. In these plots, colored dots indicate a temporal feature of the CR (onsets, maxima, offsets) and the blue line indicates US onset. Pause maxima occurred shortly before US onset. Pause onsets always preceding the expected US and pause offsets always succeeding the US onset is due to some recorder units encoding low time steps and others encoding time-steps longer than the ISI. Bars represent standard errors measured over ten simulations in which τ_*write*_, τ_*read*_, *R*_*i*_, and *c* varied randomly.

Next, we trained the cell on a full battery of ISIs ranging from 150 to 500 ms in increments of 50 ms. All other training parameters were fixed from the first 200 ms ISI simulation. For this wide range of ISIs, the cell learned pauses with well-timed onsets, maxima, and offsets (Figure 5). The timing of these pause features mimics those found in experimental data (Johansson et al., 2014). By design, the model cannot learn ISIs < 100 ms.

### 3.2. Simulation 2: The cell response is invariant to probe CS structure

**Figure 6:**
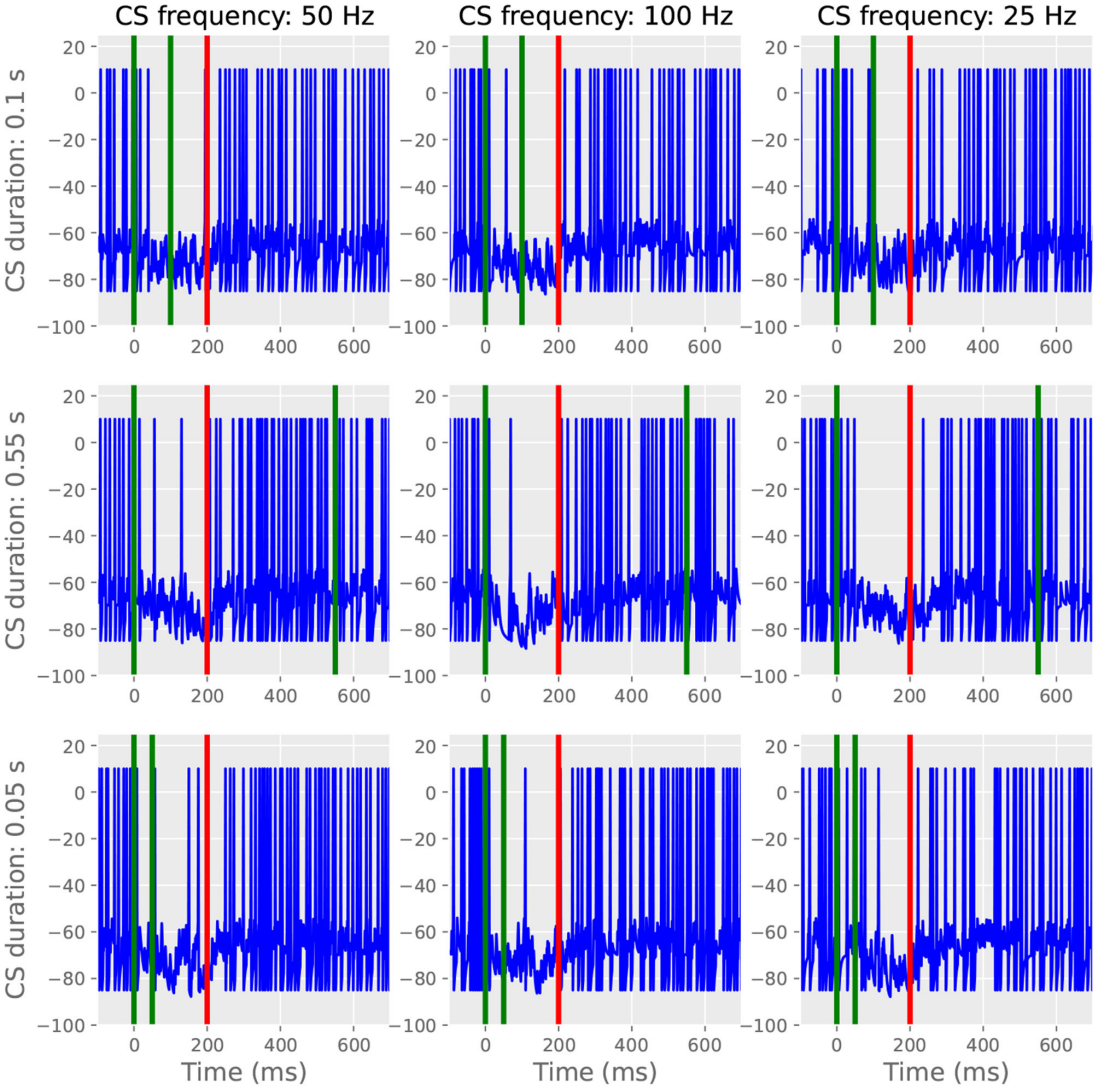
The CR is invariant to probe duration and frequency. For all panels, green lines indicate CS onset and offset and red lines indicate US onset. Left and middle column; when using 50 or 100 Hz, changing the length of the probe CS to 100, 550, or 50 ms does not affect the temporal features of the CR. The responses are also similar when using different frequencies. Right column: less evident pause responses with 25 Hz CS stimulation.

Next, we stimulated the trained cell with probe CSs enduring for lengths different from those used during training. Like in Johansson et al. (2014), we trained a cell with a 100 Hz 200 ms CS and then probed it with CSs that were altered in either duration or frequency (Figure 6). Independent of probe CS duration and frequency, the cell still produced a well-timed CR, albeit possibly faring worse with the lowest frequency tested (right column, not tested *in vivo*). The response invariance arises from the model's switch mechanism: a small amount of CS stimulation is sufficient to trigger the reading of the stored pause information, during which subsequent afferent parallel fiber activity has little effect. This simulation demonstrates that the model satisfies the computational constraint stipulated in the introduction: the learned pause is expressed on probe trials, independent of CS duration. Seemingly less robust responses with the lowest frequency tested is presumably due to insufficient input summing to trigger reading.

### 3.3. Simulation 3: The pause is extinguished over many CS-only trials

**Figure 7:**
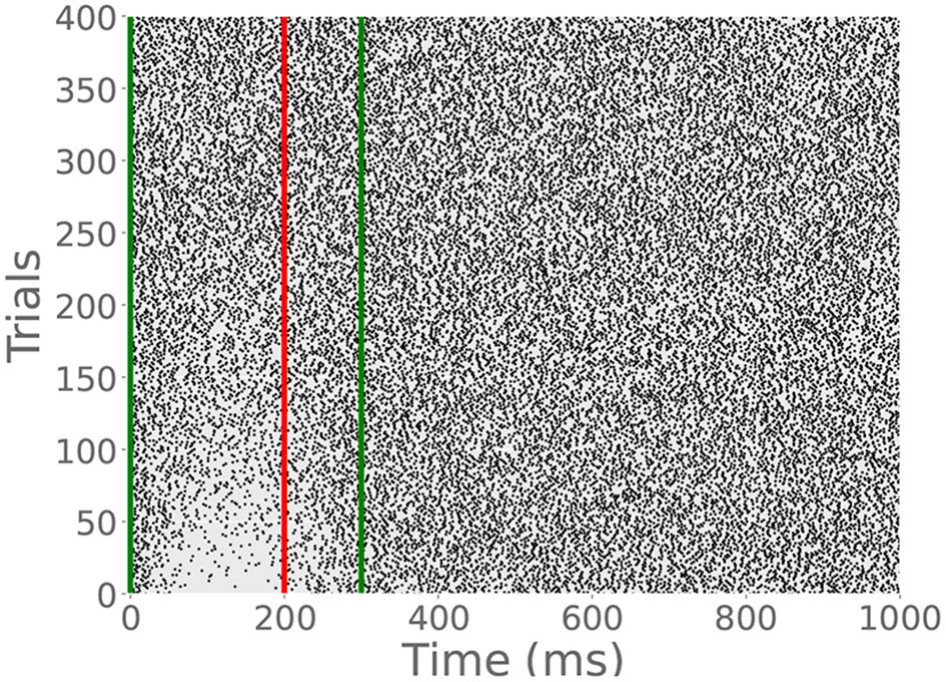
CR extinction. Green lines indicate CS onset and offset, and the red dashed line indicates expected US onset, though no US stimulation was provided in this simulation. After many CS-only trials, the CR disappears, though the archive is not empty. It has simply been reduced enough to permit tonic spiking.

Because the read module gradually depletes the archive, many CS-only trials will eventually extinguish the learned pause response. To demonstrate this behavior, we provided a cell previously trained on a 200 ms ISI with an additional 400 CS-only trials. The raster plot in Figure 7 shows the gradual extinction of the pause. This is in line with experimental data which shows the timescale of extinction is typically less than or equal to that of acquisition (Jirenhed et al., 2007).

### 3.4. Simulation 4: The cell can learn multiple pauses if trained with different ISIs on interleaving trials

**Figure 8:**
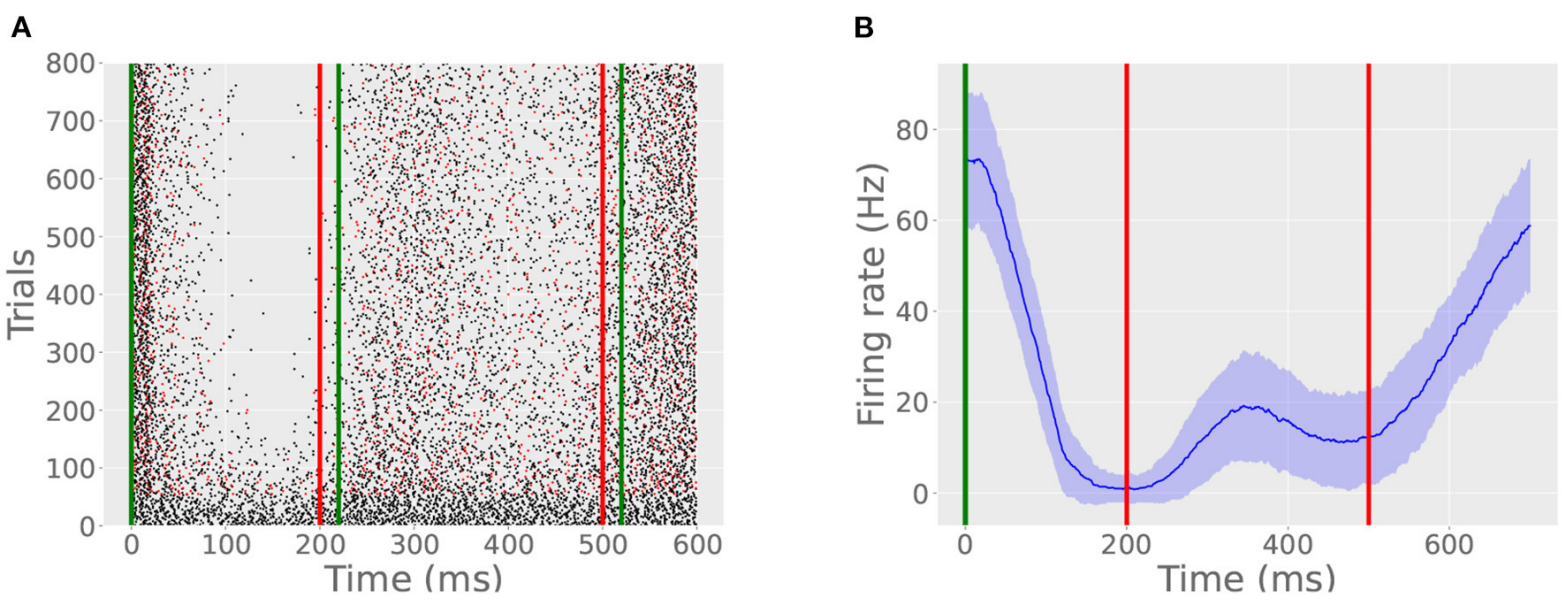
A bimodal pause. **(A)** Green lines indicate CS onset and offset, the red line indicates US onset, and red spikes indicate probe trails. In this simulation, the cell learned two ISIs, one timed to 200 ms and another timed to 500 ms. The CR consists of two pauses with an intervening resumption of spiking between 200 and 400 ms. **(B)** The bimodal CR is evident from the average firing rate plot, created in the same manner as described in Simulation 1.

Jirenhed et al. (2017) found that interleaving trials with different ISIs teaches the Purkinje cell two CRs so that, given one probe CS, the cell will pause twice. Our model Purkinje cell reproduces this behavior (Figure 8) in the case of interleaved 200 and 500 ms ISI trials with a 30 s ITI. However, in this simulation, both pauses were weaker and took more time to express using the same detection criterion. This is because only half of the trials contribute to the learning of each ISI, and those trials which do not contribute to one ISI still consume the archive by engaging the read module. As a result, experimental time to acquisition is more than twice that of the original case, adjusted for ITI. Presumably, this paradigm could be generalized to more than 2 pauses, as long as the modes of the archive are well-separated enough to allow for spiking resumption between the constituent pauses.

Simulations 1–4 have been performed experimentally *in vivo*. For simulations 5 and 6, we ran experiments which, to our knowledge, have not been performed experimentally and are thus purely predictive.

### 3.5. Simulation 5: The cell cannot learn multiple pauses if trained with different ISIs on the same trials

**Figure 9:**
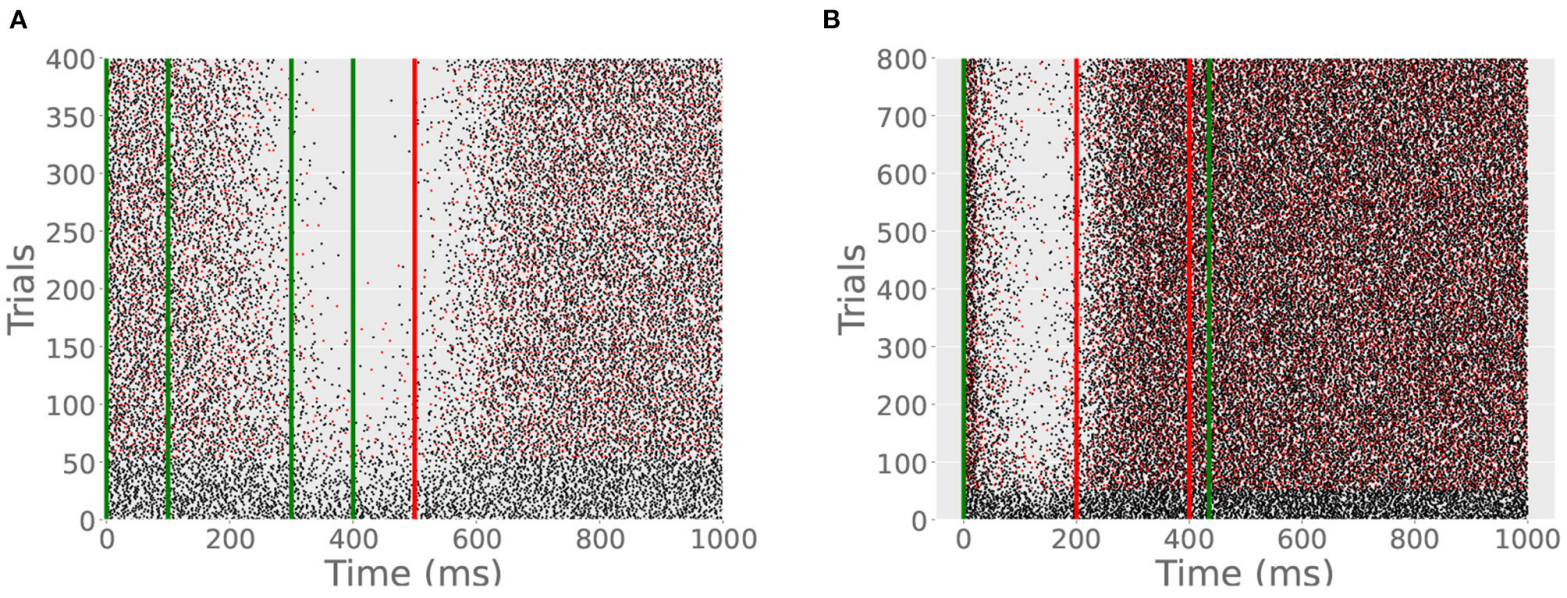
**(A)** With 2 CSs per trial, only one CR develops. Green lines indicate the CS onsets and offsets; red line indicates US onset. A CR appears beginning at around 400 ms and terminates shortly after the US. If the cell had learned the second ISI, the pause would begin later in the trial. **(B)** With 2 USs per trial, only one CR develops. Red lines indicates the first and second US onsets. Spiking resumes shortly after the first US since only the shorter ISI is learned.

Next, we tested the outcome of training the model cell with different ISIs on the same trials. This can be done in two ways. First, we trained the model cell with two CSs on each trial. One CS began at *t* = 0 and ended at *t* = 100 ms; another began at *t* = 300 ms and ended at *t* = 400 ms. US onset was at 500 ms and lasted for 20 ms. Hence, the cell was effectively exposed to two ISIs on each trial, 200 and 500 ms. Like previous simulations, both CSs were 100 Hz and the US was 500 Hz. The ITI was 15 s. Whereas, interleaving two ISIs across trials was found to produce two pause responses in Simulation 4, providing the cell with two CSs on each trial produced only one in an experiment lasting 800 trials (Figure 9A). The cell only learns the longer ISI since the refractory period on the write switch prevents it from detecting the second CS (Figure 5).

Next, we stimulated the cell with two USs on each trial, one at 200 ms and another at 400 ms, both lasting for 20 ms at 500 Hz. The CS extended to the offset of the second US. The simulation lasted for 400 trials with an ITI of 15 s. The cell learned one pause, timed to the shorter ISI (Figure 9B). Similar to the double CS experiment, the cell becomes insensitive to further US stimulation after the first US onset. The difference between the two protocols is that with two CSs the cell learns the longer of the two effective ISIs and with two USs the cell learns the shorter effective ISI.

### 3.6. Simulation 6: Trials to acquisition depends on ISI and ITI

*In vivo*, longer ISIs are said to take longer to learn (Jirenhed and Hesslow, 2011a). However, at the level of the Purkinje cell, to our best knowledge, this has only been studied while keeping the ITI constant. Our model predicts that the number of trials until pause acquisition is affected by several factors. In our model longer ISIs are harder to learn since noise during writing tends to flatten the histograms added to the archive, which consequently takes more trials to build up enough inhibition to cancel spiking. In contrast, short ISIs yield highly peaked histograms and therefore speed up learning. Further, long ITIs tend to speed up learning since they allow the reserve to replenish fully and release a maximum number of recorder units during subsequent reading. Short ITIs, on the other hand, do not allow for full replenishment, so that fewer recorder units are released and the archive grows more slowly.

**Figure 10:**
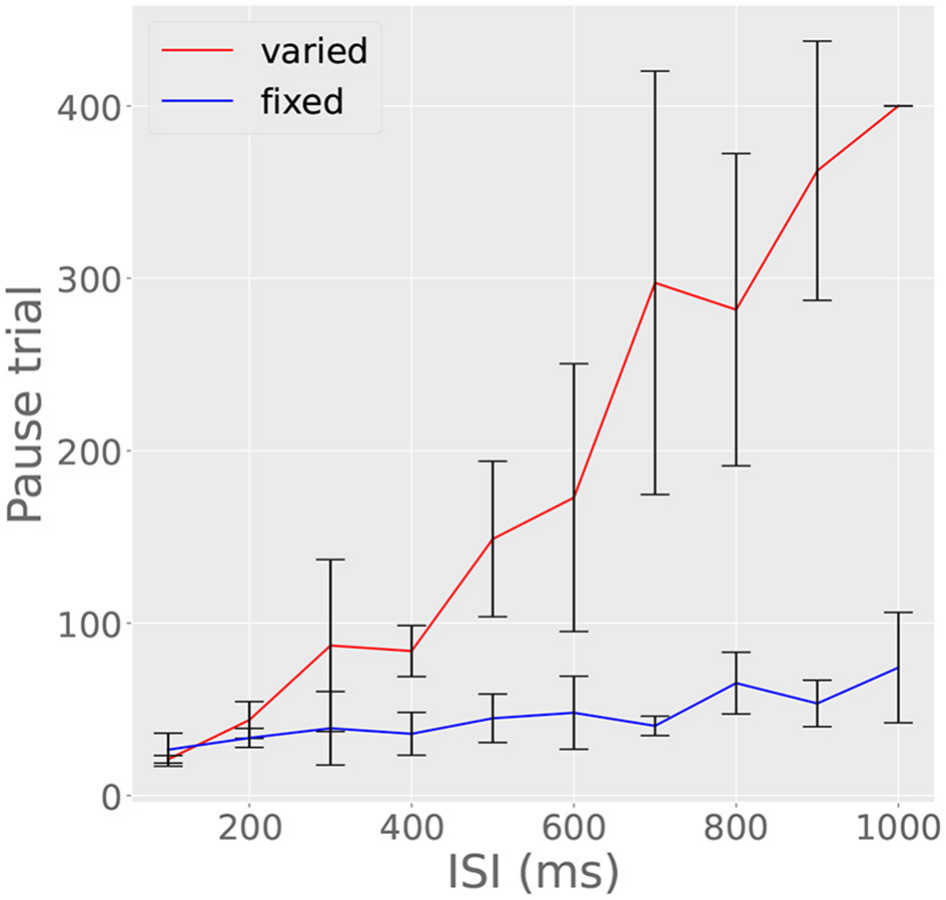
ITI affects trials to acquisition. Red line indicates trials to acquisition in experiment in which ISI varied and ITI was fixed at 15 s; blue line indicates trials to acquisition in experiments in which the ISI varied, but ITI was fixed at 80 times the ISI. Otherwise, the red line increases linearly due to the effect of noise on long ISIs and the inability of the reserve to completely replenish in 15 s. The blue line, on the other hand, is constant, since the increased noise of long ISIs is compensated for an increased ITI. Bars represent standard errors measured over ten simulations in which τ_*write*_, τ_*read*_, *R*_*i*_, and *c* varied randomly.

To investigate how these factors precisely affect our model cell, we ran two parallel experiments. In one experiment, we fixed the ITI at 15 s and trained the cell on ISIs ranging from 100 to 1,000 ms in steps of 10 ms. For the other experiment, we used the same range of ISIs, but also varied the ITI so that ITI/ISI = 80 for each ISI. In the first experiment, trials to acquisition increased monotonically and approximately linearly with ISI (Figure 10, red line). However, when the ITI/ISI ratio was held constant, the hindering effect of noise due to increasing ISI was canceled out by the accelerating effect of the increased ITI, resulting in a flat trials-to-acquisition curve (Figure 10, blue line). This effect has not yet been tested in the Purkinje cell, though it has been observed in other behavioral experiments (Gibbon, 1977). The finding illustrated in Figure 10 suggests that trials to acquisition T is given roughly by T = 8,000 (ISI/ITI).

## 4. Discussion

This purely formal model recapitulates the behavior of the Purkinje cell during eyeblink conditioning. The model cell learns a pause response with characteristically timed onset, maximum and offset. Extinction of the pause is also similar to *in vivo* data. Further, the model obeys the two desiderata stipulated in the introduction: the POT representation makes no use of the upstream network and pause response learning and expression machinery is gated by a switch. Obeying these criteria is what permits the model to replicate more of the data. The obvious limitation of the model is that it is agnostic as to possible biochemical implementations. Yet, these proof-of-principle simulations could inspire electrophysiological experimentation and biochemical modeling.

One of the greatest strengths of this model is that, to our knowledge, it is the first to capture the fact that the Purkinje cell pause response, once learned, is remarkably robust to changes in the parameters of the CS. As noted in the introduction, the time course of the response is determined by the initial part (< 20 ms) of the CS. Once an animal has been trained with a particular CS duration (e.g., 400 ms), a very brief CS or sometimes even a single impulse delivered to the mossy fibers, can elicit a well-timed Purkinje cell pause (Jirenhed and Hesslow, 2011b). Network timing models would need the dramatically different inputs to the network to cause similar patterns of activity in it. Further, even short-lasting input delivered directly to the immediate Purkinje cell afferents, bypassing the network, is sufficient to elicit the full pause response (Johansson et al., 2014). Our model recapitulates this phenomenon well since CS activity is not directly translated into Purkinje spiking, but rather acts as a trigger to retrieve stored information. While we cannot point to a specific biochemical implementation we here show an example of what kind of mechanism could explain the data in principle.

Additionally, our model makes several non-obvious and testable predictions. The main reason that the first two predictions are important to test empirically is that we believe that the results would differentiate between our type of model and other current models. First, if the Purkinje cell is presented with complex inputs, effectively experiencing two different ISIs on the same trial, it will only learn one ISI. In simulation 5, the cell only learns the longest ISI when there are two sequential CSs on the same trial. In the first test there is parallel fiber input between *t* = 0 and *t* = 100 ms, and then between *t* = 300 ms and t=400 ms. The US onset is at *t* = 500 ms. Our model cell learns the longer trace ISI of 500 ms. This is presumably not the outcome of such a protocol in customary models where the POT representation lies in granule cell firing. Our interpretation of these models is that the outcome would be either no learning or possibly some learning of the shorter ISI if there is some sustained granule cell firing after the second CS. In the second test there is parallel fiber input between *t* = 0 and *t* = 400 ms with one US at *t* = 200 ms and a second US at *t* = 400 ms. Here, our model cell only learns the shorter ISI (200 ms) even when the CS extends to the later US. Again, this contrasts with established models where our interpretation is that the Purkinje cell will learn both ISIs.

Finally, our model predicts that it could be possible to decrease the trials to acquisition for a single neuron's response by increasing the intertrial interval, a counterintuitive phenomenon hitherto only observed at the behavioral level in other conditioning paradigms (Gibbon, 1977; Gallistel and Gibbon, 2000; Ward et al., 2013). In information theory, the CS can be viewed as informative to the extent that it reduces uncertainty regarding the timing of the next US. If the ISI increases proportionally to the ITI, the ISI/ITI ratio does not change and hence the amount of information conveyed by CS onset does not change (Ward et al., 2013). It should be noted that the intertrial intervals used in the behavioral conditioning experiments where this effect occurs are often much longer, hours or even days. However, our model makes the counterintuitive prediction that the phenomenon will occur within a single conditioning session.

In contrast, by standard learning theory accounts, close temporal contiguity determines the strength of the CS-US association. Indeed, in contemporary neuroscientific models of learning where the encoding mechanism is alterations of synaptic strength, presenting fewer CS-US pairings should unequivocally weaken the associative bond, not strengthen it, as is the case in our model. We believe that this difference is an additional reason that our model could be highly informative if experimentally evaluated.

In our view, the empirical data encourages a conceptual re-imagining of cerebellar timing and we believe that the current work could inspire experimental designs to investigate intracellular mechanisms. The major outstanding limitation of the model is clearly that we do not yet know what form these mechanisms could take. For this reason, our aim is not to replace other contemporary models, but merely provide alternative thinking. At this point we can only speculate. For illustrative purposes, we would like to note that molecular computation can occur and does occur. For example, DNA incorporates sophisticated pointer arithmetic and sequences encoding complex objects (e.g., an eye) or even “syntactic” concepts (e.g., dorsal or ventral). Artificially writing data into the metabolome shows the potential power of small-molecule information systems (Kennedy et al., 2019). Moreover, Bonnet et al. (2013) have built transistors out of proteins, “transcriptors,” which can be used to build logic gates capable of allowing a cell to detect when it was impinged upon by some stimulus. Note that, for simplicity, we have supposed that recorder units can assume a continuum of states, encoding elapsed time from release. A protein-based model would suppose that recorder units can assume, say, four conformational states. Though the model's learned histogram would be much coarser in this case, it would not substantially change our model. A much smoother histogram could be learned by a model whose recorder units are polynucleotides. In this case, the number of configurations of a recorder unit would be exponential in the number of nucleotides, affording a compact, expressive symbol. There is, however, no known naturally-occurring method for storing acquired information in polynucleotides, though some artificial methods have been proposed (Kording, 2011; Choi et al., 2022).

Empirically *in vivo* data has suggested the involvement of the metabotropic glutamate receptor 7 (mGluR7) on Purkinje cell dendrites (Johansson et al., 2015), consistent with a switch-like trigger for the response. A metabotropic switch could explain the insensitivity to the temporal structure of the probe CS after conditioning and explain why there is a refractory period, because once the receptor is activated its trimeric G-protein disassociates. The split Gβγ dimer is capable of activating postsynaptic inhibition *via* Kir3 channels (Dascal, 1997; Whorton and MacKinnon, 2013), providing possible means for the hyperpolarization causing the pause response. The time course could be regulated over hundreds of ms by regulators of G-protein signaling (RGS proteins) as in the temporal regulation of photoreceptors returning to resting state (Krispel et al., 2006) and prolonged inhibition in hippocampal neurons (Xie et al., 2010). Purkinje cell modeling by Majoral et al. (2020) and Mandwal et al. (2021) incorporate these components in biochemical cascades, showing that they could replicate part of the phenomena. Until there are more empirical findings on the involved mechanisms, we here aimed at presenting a general model, exploring what is computationally feasible.

Importantly, we do not suggest that intracellular passage-of-time representations have to replace network representations (Bullock et al., 1994; Buonomano and Mauk, 1994; Medina and Mauk, 2000; Hansel et al., 2001; Yamazaki and Tanaka, 2009; Lepora et al., 2010). For instance, multiple dimensions and timescales of plasticity distributed across the cerebellar circuit (see e.g., Antonietti et al., 2017) could be concurrently operational in order to enable timed behavior.

The findings of Chen et al. (2014), Johansson et al. (2014), and Ryan et al. (2015) among others may call for a re-imagining of the neural basis of learning and memory. The model outlined above is a computational proof-of-principle for a learning mechanism inspired by this work, in the case of eye-blink conditioning. Formally, the model in many ways resembles earlier population models of timing, though here a population of neurons has been replaced with a set of abstract units within the neuron. The model makes several straightforward but non-trivial predictions, which can be tested electrophysiologically. Most importantly, a complete understanding of the Purkinje cell learning phenomenon will require insight from molecular biology, in order to illuminate the structure of the learning mechanism this paper attempts to formally describe.

## Data availability statement

The original contributions presented in the study are included in the article/supplementary material, further inquiries can be directed to the corresponding author/s.

## Author contributions

MR and JK developed the mathematical model with supervision from FJ. All authors contributed to the manuscript.
